# Contribution of Vocabulary Knowledge to Reading Comprehension Among Chinese Students: A Meta-Analysis

**DOI:** 10.3389/fpsyg.2020.525369

**Published:** 2020-10-02

**Authors:** Yang Dong, Yi Tang, Bonnie Wing-Yin Chow, Weisha Wang, Wei-Yang Dong

**Affiliations:** ^1^Department of Social and Behavioural Sciences, City University of Hong Kong, Kowloon, Hong Kong; ^2^School of Economics and Management, China University of Petroleum, Qingdao, China; ^3^Faculty of Social Sciences, Southampton Business School, University of Southampton, Southampton, United Kingdom; ^4^Department of Asian Policy Studies, The Education University of Hong Kong, Tai Po, Hong Kong

**Keywords:** vocabulary knowledge, reading comprehension, reading stage, education stage, information gap, Chinese students

## Abstract

This study investigated the correlation between vocabulary knowledge and reading comprehension. To address the correlation picture under Chinese logographical scripts, the researchers investigated the potential explanation for the correlation via Reading Stage, Information Gap, Content-based Approach, and Cognition and Creativity Theory approaches. This study undertook a meta-analysis to synthesize 89 independent samples from primary school stage to Master's degree stage. Results showed the correlation picture as an inverted U-shape, supporting the idea that vocabulary knowledge contributed a large proportion of variance on text comprehension and might also support the independent hypothesis of the impact of vocabulary knowledge on reading comprehension. In each education stage, the correlation between vocabulary knowledge and reading comprehension was independent in that it did not interact with any significant moderators. This study informed that the vocabulary knowledge not only determined text comprehension progress through facial semantic meaning identification but also suggested that the coordinate development of vocabulary knowledge, grammatical knowledge, and inference would be better in complexity comprehension task performance.

## Introduction

Reading comprehension refers to gaining meaning from the given printed text through the interaction between readers' schema knowledge retrieval and semantic cognition (Snow, 2002; Wigfield et al., 2016). Reading comprehension plays a vital role in two main learning perspectives—knowledge acquisition and cognition aptitude cultivation (Perfetti and Stafura, 2014; Silva and Cain, 2015). The Simple View of Reading (SVR) posits that the fundamental knowledge for reading comprehension is *vocabulary* knowledge (Hoover and Gough, 1990; Cromley and Azevedo, 2007). Vocabulary knowledge, regarded as the minimum semantic unit in reading comprehension and regarded as a component of linguistic comprehension, refers to a semantic schema on passage mental image cognition and single word or character semantic meaning identification (Nation, 2015; Braze et al., 2016). Large vocabulary size usually represented well-structured semantic schema and better performance in word/character meaning identification. Past studies have shown that Chinese vocabulary characters, as a representor of logographic scripts, differs from alphabetical scripts in spatial structure, grammatical knowledge, and word function (Wang et al., 2003; Elleman et al., 2009; Tong et al., 2016; Choi et al., 2017). Logographical script (e.g., Chinese characters) has a homophonic richness (Kuo and Anderson, 2006), it is not always reliable in character semantic meaning identification via phonological knowledge as alphabetical words cognition. The unique feature of Chinese characters may result in a different contribution of the vocabulary knowledge to reading comprehension. From the perspective of verbal cognition development, vocabulary knowledge may contribute more on reading comprehension activities at the higher education stage (Information Gap, Katz, 2001). In a similar vein, learning to read transited to reading to learn will be accomplished during primary school (Chall, 1987). Past studies showed that decoding contributed less variance and linguistic comprehension explained more variance in higher grades and education stage (Mol and Bus, 2011; García and Cain, 2014). However, the effect of detailed factor (e.g., vocabulary knowledge) on reading comprehension was unknown. Whether the unique effect of Chinese character characteristics (e.g., structure) would be different from other language scripts is still unclear. Therefore, the current study aims to investigate the correlation between vocabulary knowledge and reading comprehension for Chinese readers and to further investigate the potential interaction effect between selected moderators and the association between vocabulary knowledge and reading comprehension.

## Literature Review

### Vocabulary Knowledge and Reading Comprehension

Vocabulary knowledge in reading comprehension refers to a kind of knowledge that facilitates text comprehension by single, double, or more words/characters' semantic meaning identification, providing the possibility of necessary cognitive capacity for higher-level reading processes (Silva and Cain, 2015; LervAag et al., 2018). Extant literature has shown that vocabulary knowledge contributes to reading comprehension through semantic meaning identification and played a collaborator role with inference on sentence meaning comprehension (Silva and Cain, 2015; LervAag et al., 2018; Lawrence et al., 2019). High quality of word semantic meaning identification is beneficial for accurate individual word meaning retrieval (Perfetti and Hart, 2002), which establishes word-and-word unit for sentence proposition coherence (Cain et al., 2004; Braze et al., 2016). Past evidence has shown that vocabulary is significantly related to inference ability, listening comprehension, and reading comprehension (Lepola et al., 2012; Cain and Oakhill, 2014; Daugaard et al., 2017). Chinese is a kind of logographic script that is different from alphabetical script (e.g., English) in character construction (Ku and Anderson, 2003; Ramirez et al., 2010), grammatical knowledge (Bawa and Watson, 2017; Paradis and Jia, 2017), and function words sequence (Chen et al., 2016; Lee et al., 2017). Chinese characters are usually constructed by two components: the radical part usually represents the pronunciation of the character; the other side of the component represents the function of the character. The structure usually could be divided into three categories: left-right (e.g., 棋), top-down (e.g., 盛), and surround (e.g., 困). In Chinese, the restricted semantic components (e.g., time, objects, and status of the subjects) are usually inserted into the sentences rather than set at the end of the sentence or an independent component at the first part in the sentence. In particular, a single character could also be one sentence with a complete meaning [e.g., 懂 (dǒng) represents the meaning of someone understanding the whole meaning, skills, or the content that the other one mentioned]. The function and the meaning of the Chinese character are determined by the semantic meaning situation. For example, “败 (bài)” could be a verb (i.e., beat) or an adjective (lose). In the sentence “A败B,” the meaning of “败” could be win or lose; if the sentence situation shows “A” has advantages, the meaning should be win; otherwise, the meaning could be lost. Chinese characters have an omit function; the four-character idiom could represent great semantic meaning (e.g., “博大精深” represents the subject holds a great history/knowledge based on the current dialogue topic). Vocabulary knowledge contributed to reading comprehension through word recognition directly (e.g., Mezynski, 1983; McBride-Chang et al., 2005a) and through reading fluency, decoding ability, and reading rate indirectly (Hilton, 2008; Spencer and Wagner, 2018). Past studies showed that vocabulary knowledge contributed to reading comprehension process via word semantic meaning recall (semantic feature of orthographic, morphological, phonological, and pragmatic characteristics) speed and quality to achieve a mental image from the given text (Perfetti, 1985; Logan and Kieffer, 2017; Lawrence et al., 2019). However, the inconsistent results of various correlations between vocabulary knowledge and reading comprehension have been found in Chinese students, from low correlation (e.g., Cheng et al., 2017) to high correlation (e.g., Li et al., 2009). The unique effect of vocabulary knowledge on reading comprehension remains unknown among Chinese students; therefore, the role of the vocabulary knowledge effect on reading comprehension for Chinese participants requires further investigation.

### Potential Moderators Selection

The current study selects grade group, education stage, language type, and sampling area as potential moderators. Reasons are listed below.

#### Grade Group

Reading stage statement (Chall, 1987) showed that grade group would be a potential moderator on the association between vocabulary knowledge and reading comprehension. The statement showed that readers started learning to read at lower grades of the primary school and transition to reading to learn at higher grades of primary school. The higher reading stages matched higher reading cognition ability, which may have interacted with the association between vocabulary knowledge, and reading comprehension.

#### Education Stage

From the perspective of the task-oriented requirement, the Information Gap Theory (Katz, 2001) suggested that education stage—from primary school stage to Master's stage—would be a potential moderator on the association between vocabulary knowledge and reading comprehension. The higher education stage provided the higher requirement of reading comprehension tasks in word cognition, passage structure cognition, and passage main idea identification. The higher requirement of the reading comprehension task may result in a higher association between vocabulary knowledge and reading comprehension.

Empirically, grade group has been shown to have a close relationship with decoding ability, which serves as a determination factor in vocabulary knowledge (e.g., morphological knowledge on radical component meaning identification). Past studies have already shown that the association between decoding ability and reading comprehension decreased by grade group (e.g., Mol and Bus, 2011; García and Cain, 2014). According to the reading stage statement and the information gap statement on reading, the current study divided grade group into two groups. Regarding the reading stage statement, grades 1–6 of primary school were divided into lower grades of primary school, grades 1 and 2; middle grades of primary school, grades 3 and 4; and higher grades of primary school, grades 5 and 6. According to the information gap statement of reading, this study used education stage (PS: primary school, SS: secondary school, US: undergraduate stage, MS: Master's stage) to represent different grade groups.

#### Language Type

Content-based Approaches (Cloud et al., 2000) suggested that verbal cognition difficulty negatively correlated with the association between vocabulary knowledge and reading comprehension across different language scripts for readers. Past studies showed that the cognition difficulty was higher in second-language (L2) than in first-language (L1) scripts. In addition, it was confirmed that morphological knowledge made a higher contribution to logographic scripts cognition than phonological knowledge (Yeung et al., 2011; Ruan et al., 2018). In contrast, phonological knowledge made a higher contribution to alphabetical scripts cognition than to logographical scripts (Seidenberg, 2011). The current study selected Chinese students as participants; thus, the cognition difficulty might be higher in alphabetical scripts comprehension than in logographical scripts comprehension. Therefore, the language type may interact with the association between vocabulary knowledge and reading comprehension.

#### Sampling Area

Cognition and Creativity Theory (Runco, 2007) suggested that verbal ability application in reading comprehension was affected by visual and auditory cognition. Mainland China, Hong Kong, and Taiwan have different writing systems and oral language systems in Chinese academic studies (e.g., Siok and Fletcher, 2001; McBride-Chang et al., 2005b). Regarding the writing system, mainland China uses a simplified script while both Hong Kong and Taiwan use traditional script. The differences mainly come from the number of strokes (the simplified version has ~22.5% fewer strokes than the traditional version has) and characters' structure complexity (traditional script is more complex). In addition, the pronunciation, grammatical knowledge, and sentence construction are very different between Mandarin (used in mainland China and Taiwan) and Cantonese (used in Hong Kong). The complexity of words impacts reading comprehension performance (Filippi et al., 2015; LervAag et al., 2018).

### Relevant Meta-Analysis Studies Between Vocabulary and Reading Comprehension

In the last three decades, a few studies investigated the effect of vocabulary knowledge on reading comprehension. These mainly adopt two mainstream approaches to synthesize the effect size between vocabulary knowledge and reading comprehension. The majority of studies focus on vocabulary knowledge intervention effect on reading comprehension (e.g., Elleman et al., 2009; Marulis and Neuman, 2010; Dexter and Hughes, 2011), providing each effect size for specific intervention programs. The second group reflects the correlation between vocabulary knowledge and reading comprehension. However, past correlational meta-analytic studies have three main limitations. First, such studies (e.g., Jeon and Yamashita, 2014) only included a small number of empirical studies, which may not represent the real correlation between vocabulary knowledge and reading comprehension. In addition, the study by Jeon and Yamashita (2014) did not provide any convincing association results, because the heterogeneity problem and the outliers were not removed. Second, past studies show limitations in participants' selection. For example, Kudo et al. (2015) reported the correlation between vocabulary knowledge and reading comprehension in readers with learning difficulties only. Finally, a few studies provided the correlation picture on logographical scripts' characters in which semantic meaning could be defined via morphemes.

### The Current Study

The current study investigates the picture between vocabulary knowledge and reading comprehension for Chinese students from primary education stage to Master's education stage. Specifically, this study investigates the possible interaction effect explanations for the association between reading comprehension and vocabulary knowledge in Chinese readers from the reading stage, information gap, content-based approaches, and cognition and creativity theory perspectives. Moreover, the interaction effect of education stage, grade group, language type, and sampling area with the association between vocabulary knowledge and reading comprehension is also examined. Under the guidelines of *PRISMA*, the current study selects the most recent 20 years of empirical studies as materials, investigating the correlation between vocabulary knowledge and reading comprehension in Chinese students.

## Methods

### Literature Base

This study selected potential materials from different databases. To avoid any misunderstanding of the scripts, the authors selected the materials written in Chinese and English only. The Chinese materials were selected from the CNKI database, which included all possible academic empirical studies written in Chinese. Empirical studies written in English were selected from PsycINFO, ERIC, and Pro-Quest Dissertations and Theses. Two groups of key terms were used to search the empirical studies. Group 1 refers to vocabulary knowledge, including vocabulary^*^, vocabulary knowledge^*^, breadth of vocabulary^*^, and depth of vocabulary^*^. The second group refers to reading comprehension, including sentence comprehension^*^, paragraph comprehension^*^, passage comprehension^*^, text comprehension^*^, reading ability^*^, reading performance^*^, and comprehension^*^. All searched materials were published in the last 20 years (1998–2018).

### Inclusion Criteria

All selected empirical studies (articles, dissertations, and conference paper) have to meet all the following criteria: (a) sample size over 30; (b) empirical studies and non-opinion studies; (c) provided exact reading comprehension scores; (d) participants were Chinese students; (f) Chinese was L1 for participants; (g) reading comprehension measurement reported sentence comprehension scores or passage comprehension scores; and (h) provided enough indicators for effect size calculation. Regarding correlation indicator, this study included correlation (*r*) and percentage of variance (*R*^2^) in reading comprehension accounted for by vocabulary knowledge.

**Figure 1:**
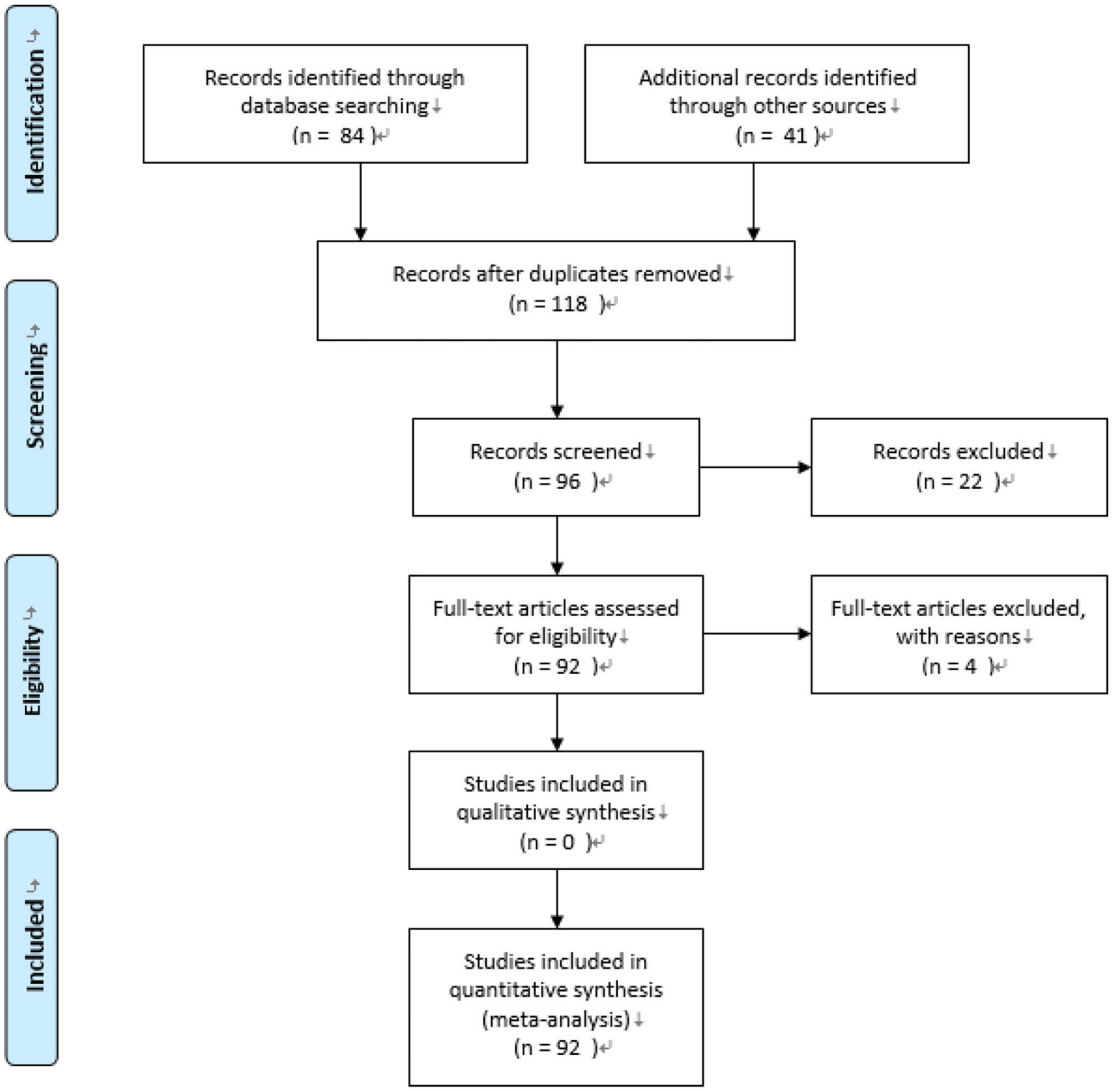
Flow chart for material selection.

In addition, those studies with composite measurement of reading skills (e.g., vocabulary plus reading comprehension and reading plus listening comprehension) were removed in order to ensure that the effect size only reflected the correlation between reading comprehension and vocabulary knowledge. Moreover, both vocabulary knowledge and reading comprehension should be measured at the same time from the same sample because the current study tries to report the concurrent correlation between vocabulary knowledge and reading comprehension. Detailed information of potential studies search was provided in Figure 1.

### Coding Process

Two coders coded the following information independently: (a) year of publication, (b) first author, (c) sampling area, (d) sample size, (e) grade group, (f) education stage, (g) language type, and (h) effect size of the correlation between vocabulary knowledge and reading comprehension. If the data were absent from the original materials, the coders emailed the authors for information. Two coders removed those articles in which these eight key items were unclear.

If the selected article's participants were primary school students, to address the hypothesis of the interaction effect of the reading stage on the correlation between vocabulary knowledge and reading comprehension, the authors separated the studies as independent samples if participants came from different grade groups. To investigate the interaction effect of language type on the association between vocabulary knowledge and reading comprehension, the authors separated the studies as independent samples if one article provided the following two correlations—the first one was between L1 vocabulary knowledge and L1 reading comprehension, and the second one was between L2 vocabulary knowledge and L2 reading comprehension. This study removed those correlation effect sizes where the vocabulary knowledge and reading comprehension came from different language scripts, specifically the effect size between L1 vocabulary knowledge and L2 reading comprehension and the effect size between L2 vocabulary knowledge and L1 reading comprehension. Otherwise, if one article provided more than one available effect size, they were subjected to robust variance estimation (Hedges et al., 2010) for effect size estimation, ensuring that each independent sample only provided one effect size for further meta-analysis. The intercoder agreement for both study characteristics and outcome variables was 95% across meta-analyses, and all discrepancies between coders came from the sampling area. The authors solved this problem by removing those articles in which the sampling area was mixed—for example, the participants came from both mainland China and Hong Kong and the correlation effect size was not clear for either sampling area.

### Meta-Analytic Procedures

This study followed standard analytic procedures as claimed in *PRISMA* (Moher et al., 2011). All correlation indicators were entered into Comprehensive Meta-analysis for Fisher's *z* calculation. This study selected Fisher's *z* because *z* followed asymmetrical distribution (Borenstein et al., 2009). To interpret the effect size, the values of Fisher's *z* were 10, 31, and 55, to be interpreted as small effect size, moderate effect size, and large effect size, respectively (Cohen, 1988).

To be conservative, this study applied indicators from the random-effect model, which includes the value of Fisher's *z*, variance, *Q*-value, and 95% confidence interval (CI). Fisher's *z* could be interpreted as significant when 95% CIs do not cross zero (Hedges and Pigott, 2004). Then, meta-regression was applied for moderator analysis when *Q* reached a level of significance. This study also examined sensitivity analysis through randomly removing one sample from the list. Furthermore, Orwin's safe number, funnel plot through trim-and-fill approach, *p*-value of Begg's rank correlation test, and Egger's regression intercept test were reported to address publication bias.

To compare the effect sizes between each group, the authors calculated δ for further analysis: δ = *Diff* /*SE, Diff* = Fisher's *z*_1_ – Fisher's *z*_2_, *SE* = Sqrt (Variance *z*_1_ + Variance *z*_2_), if |δ| ≥ 1.96. They interpret the result to have significant difference (*p* < 0.05).

## Results

### Descriptive Statistics

Detailed information of selected studies were shown in Table 1. Three outliers from primary school grades' list were removed due to an effect size of over 3.5 standard deviation (García and Cain, 2014): Cheng and Wu (2017) from the lower primary grades' list and Chen (2015) and Chen at al. (2018) from the higher primary grades' list. The remaining 81 studies included in the meta-analysis represented a total of 10,668 participants obtained from 89 independent samples. Of these, 29 samples (*n* = 4,672) reported the correlation between vocabulary knowledge and reading comprehension for primary school students. In particular, 17 samples (*n* = 2,400) reported the correlation in lower primary grades, 6 samples (*n* = 1,019) reported the correlation in middle primary grades, and 6 samples (*n* = 1,253) reported the correlation in higher primary grades. Furthermore, 21 samples (*n* = 3,122) reported the correlation between L1 vocabulary knowledge and L1 reading comprehension, and 8 samples (*n* = 1,550) reported the correlation between L2 vocabulary knowledge and L2 reading comprehension.

**Table 1:** Descriptive information of the selected studies.

**No**.	**First author, year of publication**	**Sampling area^**a**^**	**Grade group^**b**^**	**Education stage^**c**^**	**Language type^**d**^**	**Sample size**	**Effect size**	***SE***
1	Zhang H. (2013)	MC	NA	SS	2	108	0.79	0.10
2	Lu and Zhang (2015)	MC	NA	SS	2	108	0.79	0.10
3	Zhang and Koda (2018)	MC	NA	US	2	195	0.50	0.07
4	Li (2012)	MC	NA	US	2	115	0.54	0.09
5	Shen (2014)	MC	NA	US	2	68	0.47	0.12
6	Wang (2010)	MC	NA	US	2	146	0.55	0.08
7	Zhang D. (2012)	MC	NA	MS	2	190	0.29	0.07
8	Liao (2012)	MC	NA	SS	2	44	0.56	0.16
9	Lam et al. (2012)	Others	L	PS	2	80	0.60	0.11
10	Huang (2003)	MC	NA	US	2	90	0.76	0.11
11	Zhang (2011)	MC	NA	US	2	33	0.40	0.18
12	Cheng et al. (2017)	MC	L	PS	1	149	0.32	0.08
13	Chen et al. (2014)	MC	NA	US	2	135	0.63	0.09
14	Wang (2007)	MC	NA	US	2	52	0.64	0.14
15	Wang (2013)	MC	NA	US	2	60	0.62	0.13
16	Wang (2011)	MC	NA	US	2	132	0.43	0.09
17	Gong (2006)	MC	NA	SS	2	60	0.80	0.13
18	Jin (2011)	MC	NA	US	2	141	0.54	0.09
19	Zhang M. (2012)	MC	NA	US	2	50	0.55	0.15
20	Gao (2012)	MC	NA	US	2	74	0.52	0.12
21	Zong (2017)	MC	NA	SS	2	51	0.71	0.14
22	Ho et al. (2012)	HK	H	PS	2	388	0.41	0.05
23	Liu (2010)	MC	NA	US	2	64	0.59	0.13
24	Liu (2006)	MC	NA	US	2	65	0.59	0.13
25	Zhu and Li (2014)	MC	NA	US	2	115	0.51	0.09
26	Guo and Roehrig (2011)	MC	NA	US	2	278	0.49	0.06
27	Zhang et al. (2012)	HK	L	PS	1	164	0.47	0.08
28	Gan and Qiu (2012)	MC	NA	US	2	47	0.56	0.15
29	Tan (2005)	MC	NA	US	2	106	0.56	0.10
30	Yue (2009)	MC	NA	US	2	107	0.56	0.10
31	Yan et al. (2007)	MC	NA	US	2	118	0.60	0.09
32	Hou (2016)	MC	NA	US	2	212	0.64	0.07
33	Tian (2012)	MC	NA	US	2	40	0.80	0.16
34	Zou (2006)	MC	NA	US	2	69	0.59	0.12
35	Liu (2012)	MC	NA	US	2	87	0.64	0.11
36	Liu (2005)	MC	NA	US	2	128	0.58	0.09
37	Lin W. (2015)	MC	NA	SS	2	60	0.82	0.13
38	Zhou (2015)	MC	NA	SS	2	32	0.77	0.19
39	Che (2017)	MC	NA	US	2	102	0.31	0.10
40	Chen et al. (2018)	TW	H	PS	1	164	0.87	0.08
41	Ye and Geng (2013)	MC	L	PS	1	194	0.58	0.07
42	Chang (2010)	MC	H	PS	2	175	0.59	0.08
43	Zhang (2017)	Others	M	PS	1	265	0.53	0.06
44	Lei and Xiao (2017)	TW	NA	US	2	53	0.70	0.14
45	Deng (2014)	MC	NA	US	2	70	0.62	0.12
46	Shen and Wei (2011)	MC	NA	US	2	68	0.78	0.12
47	Zhou et al. (2016)	MC	L	PS	1	192	0.61	0.07
48	Zhang H. (2016)	MC	L	PS	1	123	0.41	0.09
49	Qiu (2011)	MC	NA	SS	2	92	0.74	0.11
50	Gao (2011)	MC	NA	US	2	60	0.73	0.13
51	Zhang and Koda (2013)	MC	H	PS	2	245	0.46	0.06
52	Zhang and Zhao (2011)	MC	NA	MS	2	190	0.34	0.07
53	Wang (2006)	MC	NA	US	2	239	0.53	0.07
54	Zou (2011)	MC	NA	US	2	69	0.59	0.12
55	Zhang and Koda (2012)	MC	NA	MS	2	130	0.22	0.09
56	Liu (2009)	MC	NA	US	2	73	0.38	0.12
57	Li (2008)	MC	NA	US	2	53	0.69	0.14
58a	Wu et al. (2009)	MC	L	PS	1	154	0.56	0.08
58b	Wu et al. (2009)	MC	M	PS	1	146	0.58	0.08
59	Zou and Guo (2008)	MC	NA	US	2	39	0.47	0.17
60	Qi (2014)	MC	NA	US	2	63	0.55	0.13
61	Zhang J. (2013)	MC	NA	US	2	53	0.72	0.14
62	Xia (2016)	MC	NA	US	2	35	0.32	0.18
63	Luo (2009)	MC	NA	SS	2	124	0.78	0.09
64	Bian (2017)	MC	NA	US	2	191	0.47	0.07
65	Chang et al. (2014)	MC	NA	US	2	78	0.35	0.12
66	Li et al. (2009)	MC	L	PS	1	140	0.55	0.09
67a	Li et al. (2012)	HK	H	PS	1	141	0.38	0.09
67b	Li et al. (2012)	HK	L	PS	2	141	0.76	0.09
68	Yan (2009)	MC	NA	US	2	76	0.55	0.12
69	Lin X. (2015)	MC	NA	SS	2	115	0.65	0.09
70	Wu (2011)	MC	M	PS	1	78	0.66	0.12
71	Zhu (2016)	MC	NA	SS	2	56	0.65	0.14
72	Liu (2008)	MC	NA	US	2	62	0.52	0.13
73	Zhang D. (2012)	MC	NA	MS	2	130	0.22	0.09
74	Ma and Lin (2015)	TW	NA	US	2	124	0.47	0.09
75	Tsai et al. (2010)	TW	NA	US	2	271	0.50	0.06
76	Zhang and Koda (2014)	MC	H	PS	1	245	0.53	0.06
77a	McBride-Chang et al. (2005a)	HK	L	PS	1	100	0.37	0.10
77b	McBride-Chang et al. (2005b)	MC	L	PS	1	100	0.38	0.10
78	Chen (2015)	TW	H	PS	1	164	0.84	0.08
79a	Chik et al. (2012)	HK	H	PS	1	59	0.47	0.13
79b	Chik et al. (2012)	HK	L	PS	1	119	0.45	0.09
80	Wang et al. (2006)	Others	L	PS	1	64	0.50	0.13
81	Cheng and Wu (2017)	MC	L	PS	1	149	0.39	0.08
82a	Cheng et al. (2016)	MC	L	PS	1	149	0.41	0.08
82b	Cheng et al. (2016)	MC	L	PS	1	127	0.39	0.09
83a	Siu and Ho (2015)	HK	L	PS	1	202	0.54	0.07
83b	Siu and Ho (2015)	HK	L	PS	2	202	0.48	0.07
83c	Siu and Ho (2015)	HK	M	PS	1	211	0.45	0.07
83d	Siu and Ho (2015)	HK	M	PS	2	211	0.46	0.07
84	Zhang D. (2016)	Others	M	PS	2	108	0.54	0.10

a HK, Hong Kong; MC, Mainland China; TW, Taiwan; Others, Sampling area was not China;

b L, grade 1 and grade 2 of primary school; M, grade 3 and grade 4 of primary school; H, grade 5 and grade 6 of primary school;

c PS, primary school stage; SS, secondary school stage; US, undergraduate stage; MS, Master's stage;

d *1, first language; 2, second language*.

Eleven (11) samples (*n* = 850) reported the correlation effect size in secondary school students. All 11 samples reported the correlation between L2 vocabulary knowledge and L2 reading comprehension. Next, 45 samples (*n* = 4,506) reported the correlation effect size in undergraduate students. All 45 samples reported the correlation between L2 vocabulary knowledge and L2 reading comprehension. Four samples (*n* = 640) reported the correlation in Master's students. All four samples reported the correlation between L2 vocabulary knowledge and L2 reading comprehension.

Eleven (11) samples (*n* = 1,938) reported Hong Kong students' correlation between vocabulary knowledge and reading comprehension. A further 72 samples (*n* = 7,914) reported mainland China students' correlation between vocabulary knowledge and reading comprehension. Four samples (*n* = 517) reported the correlation between vocabulary knowledge and reading comprehension for those Chinese students who lived in other countries. Five samples (*n* = 776) reported Taiwan students' correlation between vocabulary knowledge and reading comprehension.

### Meta-Analysis

As shown in Table 2 the overall correlation effect size between vocabulary knowledge and reading comprehension was nearly large (*z* = 0.54, *p* < 0.001). The *Q*-value was significant (*Q* = 204.61, *p* < 0.001). Moderator analysis showed that the education stage explained 66% (*p* < 0.001) of the variance, and the sampling area explained 10% (*p* < 0.01) of the variance. Language type did not have a significant interaction effect with the correlation between vocabulary knowledge and reading comprehension for Chinese participants.

**Table 2:** Meta-analysis.

	***k***	**Fisher's *z***	**Variance**	**95% CI**	***Q***	***I*^**2**^**	**Orwin's fail-safe number**	**Effect size comparison**
Overall	89	0.54	0.0002	[0.51, 0.57]	204.61^***^	55.52	885	|δ|_PS&SS_ = 5.68, |δ|_PS&US_ = 1.34, |δ|_PS&MS_ = 5.51, |δ|_SS&US_ = 5.08, |δ|_SS&MS_ = 5.69, |δ|_US&MS_ = 6.36
PS	29	0.50	0.0003	[0.46, 0.53]	34.84	19.64	259	
SS	11	0.74	0.0012	[0.67, 0.81]	4.18	<0.001	153	
US	45	0.55	0.0002	[0.52, 0.58]	39.97	<0.001	447	
MS	4	0.28	0.0016	[0.20, 0.35]	1.77	<0.001	19	

*** *p < 0.001, PS, primary school; SS, secondary school; US, undergraduate stage; MS, Master's stage*.

**Figure 2:**
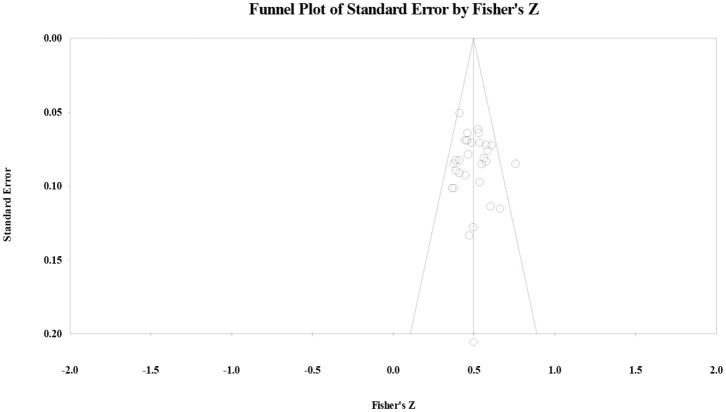
Funnel plot of the correlation effect size between vocabulary knowledge and reading comprehension for primary school students.

To further address the hypothesis from the Information Gap statement and the Reading Stage statement, following the application of data-driven approach under the guidance of *PRISMA*, the authors further examined the correlation between vocabulary knowledge and reading comprehension in each education stage through heterogeneity analysis. Regarding primary school, the effect size was 50 (*p* < 0.001) and the *Q*-value was 34.84 (*p* > 0.10, *I*^2^ = 19.64). The publication bias test showed that Orwin's fail-safe number was 259, the Tau value for Begg's rank correlation test was 03 (*p* > 0.10), and Egger's regression intercept was 49 (*p* > 0.10). The funnel plot showed that effect size had a symmetry distribution (Figure 2), indicating that the correlation effect size for primary school students did not have significant publication bias. Results suggested that reading stage statement did not have a significant interaction effect with the correlation between vocabulary knowledge and reading comprehension in primary school. Regarding sensitivity analysis, the authors randomly removed one study from the list. The result was similar, indicating that the results had higher reliability.

**Figure 3:**
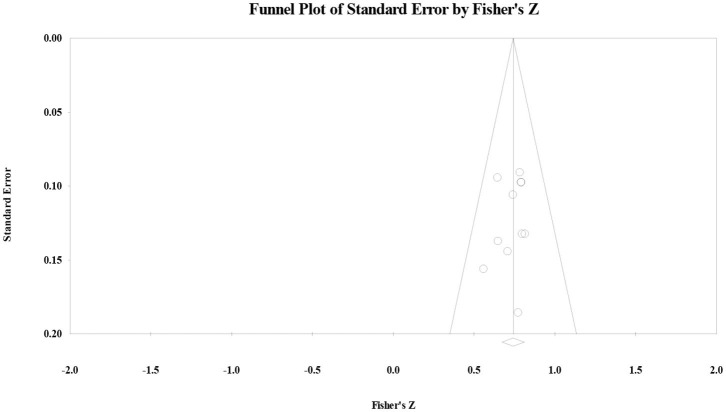
Funnel plot of the correlation effect size between vocabulary knowledge and reading comprehension for secondary school students.

Regarding secondary school, the effect size was 74 (*p* < 0.001) and the *Q*-value was 4.18 (*p* > 0.10, *I*^2^ < 0.001). The publication bias test showed that Orwin's fail-safe number was 153, the Tau value for Begg's rank correlation test was 22 (*p* > 0.10), and Egger's regression intercept was 71 (*p* > 0.10). The funnel plot showed that effect size had a symmetric distribution (Figure 3), indicating that the correlation effect size for secondary school students did not have significant publication bias. Regarding sensitivity analysis, the authors randomly removed one study from the list. The result was similar, indicating that the results had higher reliability.

**Figure 4:**
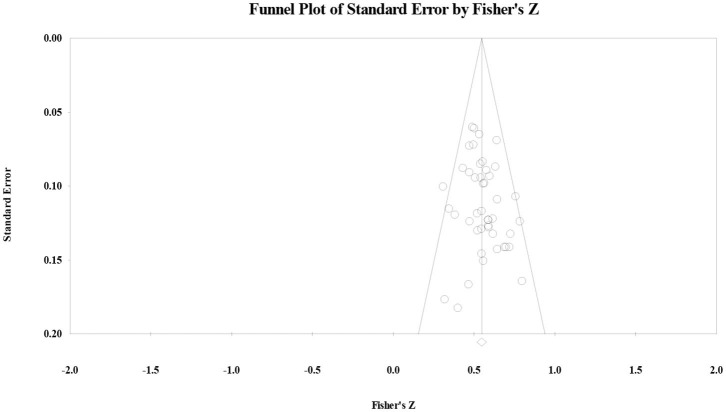
Funnel plot of the correlation effect size between vocabulary knowledge and reading comprehension for undergraduate students.

Regarding undergraduate students, the effect size was 55 (*p* < 0.001) and the *Q*-value was 39.97 (*p* > 0.10, *I*^2^ < 0.001). The publication bias test showed that Orwin's fail-safe number was 447, the Tau value for Begg's rank correlation test was 17 (*p* > 0.10), and Egger's regression intercept was 76 (*p* > 0.10). The funnel plot showed that effect size had a symmetric distribution (Figure 4), indicating that the correlation effect size for undergraduate students did not have significant publication bias. Regarding sensitivity analysis, the authors randomly removed one study from the list. The result was similar, indicating that the results had higher reliability.

**Figure 5:**
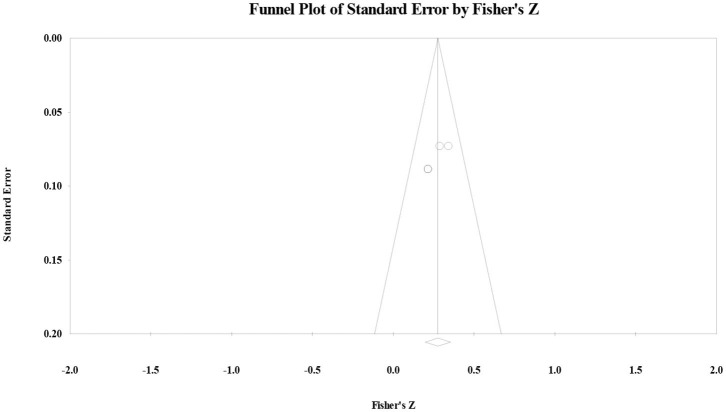
Funnel plot of the correlation effect size between vocabulary knowledge and reading comprehension for Master students.

Regarding Master's students, the effect size was 28 (*p* < 0.001) and the *Q*-value was 1.77 (*p* > 0.10, *I*^2^ < 0.001). The publication bias test showed that Orwin's fail-safe number was 19, the Tau value for Begg's rank correlation test was 60 (*p* > 0.10), and Egger's regression intercept was 6.37 (*p* > 0.10). The funnel plot showed that effect size had a symmetric distribution (Figure 5), indicating that the correlation effect size for Master's students did not have significant publication bias. Regarding sensitivity analysis, the authors randomly removed one study from the list. The result was similar, indicating that the results had higher reliability.

### Effect Size Comparison

The effect size of primary school was significantly lower than the effect size of secondary school (|δ| = 5.68, *p* < 0.001), the effect size between primary school and undergraduate was not significant (|δ| = 1.34, *p* > 0.10), and the effect size of primary school was significantly higher than the effect size of Master's students (|δ| = 5.51, *p* < 0.001). The effect size of secondary school was significantly higher than the effect size of undergraduate students (|δ| = 5.08, *p* < 0.001), and the effect size of secondary school was significantly higher than the effect size of Master's students (|δ| = 5.69, *p* < 0.001). The effect size of undergraduate students was significantly higher than the effect size of Master's students (δ| = 6.36, *p* < 0.001).

## Discussion

This study synthesized 89 independent samples to investigate the correlations between vocabulary and reading comprehension in Chinese readers from primary school stage to Master's stage. The overall correlation effect size was nearly large. The result is consistent with previous survey studies that have shown that vocabulary knowledge had great variance in explaining the mental image construction process via verbal cognition and semantic identification (Cain et al., 2004; Quinn et al., 2015; Gottardo et al., 2018). For example, vocabulary knowledge provides different potential semantic meanings of the target word or characters to assist readers' cognition of the adjacent coherence between words and sentences (Prior et al., 2014; Perfetti, 2017).

The correlation effect size was moderated significantly by education stage. Results showed that the interaction effect of grade group, language type, and sampling area was not significant, rejecting the possible interaction impact from the reading stage, content-based approach, and cognition and creativity statements via the link between vocabulary knowledge and reading comprehension. The correlation picture was an inverted U-shape from primary school stage to Master's stage. The tendency of the correlation was consistent with those cross-sectional studies with multiple grade groups (e.g., Chik et al., 2012) and longitudinal studies for different grade group performance surveys (Zhang et al., 2012; Siu and Ho, 2015; Cheng et al., 2016). There are three possible explanations on the significant interaction effect between education stage and the association of vocabulary knowledge and reading comprehension. Firstly, vocabulary knowledge might have an independent contribution on the reading comprehension. Previous studies argued that vocabulary knowledge contributed to reading comprehension directly due to the derived meaning of vocabulary on the mental representation construction (Ouellette and Beers, 2010; Tunmer and Chapman, 2012). Chinese readers tend to identify the semantic meaning of characters or words from morphological and orthographical coding than phonological coding (e.g., Dong et al., 2019); for example, readers tend to identify the function of the character through the radical component of characters and then ensure the pronunciation from the rest of the components, which may not determine the identifying facial and deep mental lexical meaning from the given text. Text comprehension progress relies more on semantic meaning identification on each character rather than on accurate pronunciation of the character. Semantic meaning, especially the facial semantic meaning from the given text cognition, determined the readers' mental image construction via the final global inference. Moreover, vocabulary knowledge directly impacted the process of target character or word decoding progress (Ouellette and Beers, 2010; Tunmer and Chapman, 2012), indicating that the vocabulary knowledge was an independent variable on reading comprehension cognition, which does not belong to decoding and linguistic comprehension (*The Simple View of Reading*: Hoover and Gough, 1990). Past studies confirmed that the association between decoding and comprehension decreased when the grade group increased (Mol and Bus, 2011; García and Cain, 2014); therefore, the proportion of linguistic comprehension contribution on reading comprehension should be increased. However, the current results partially match the development of linguistic comprehension, which might provide evidence for the independent effect of vocabulary knowledge development on reading comprehension. The fact that Chinese characters could be identified by the structure from students' schema could be an alternative reason. School curricular syllabus required students to enlarge vocabulary size from primary school to secondary school. Students learn new characters through retrieval decoding skills and schema knowledge and through recognizing familiar radical components and comparing the target character with previous acquired relevant characters' information; therefore, the increasing knowledge of vocabulary would have more effect on reading comprehension activities. However, since the stage of higher education, syllabus required less on students' vocabulary knowledge development but required more on students' grammatical and inference ability application; therefore, the speed and size of the vocabulary schema cognition construction development would be lower, resulting in less contribution on reading comprehension than primary and secondary education stage. Corresponding with the syllabus requirement, the interaction effect between complicated reading task in higher grade groups and the reading schema for semantic knowledge retrieval would be the third reason. Vocabulary knowledge contributed to comprehension progress via character semantic meaning identification and especially worked on facial meaning identification. From primary school to secondary school, the requirement of reading comprehension was an examination of the reading ability; the larger vocabulary knowledge base contributed to faster semantic knowledge retrieval (Wolf et al., 2000; Ecke, 2015). At the same time, the assessment of the reading comprehension task was not complicated. After graduating from secondary school, the reading knowledge schema assisted readers to imagine the mental representation from the given text. At the same time, the more complicated passage structure cognition process needed more reading knowledge (e.g., reading strategy, higher-order thinking) collaboration. When these were combined, the contribution proportion of the vocabulary knowledge decreased. For example, text reading comprehension not only needs word recognition but also needs a combination of strategies, inference ability, and other relevant factors (e.g., linguistic knowledge) to do text cognition, thereby leading to smaller correlation in higher-grade groups. An alternative reason could be reading comprehension difficulties. Readers might experience problems on global or adjacent text coherence cognition even though each word or character's meaning was well-identified (Oakan et al., 1971; Catts et al., 2016). The large effect size between vocabulary knowledge and reading comprehension informed vocabulary knowledge preliminarily provided the facial meaning on target character/word semantic identification and determined comprehension activity progress. At any education stage, the curricular design should pay more attention to students' vocabulary schema development. Moreover, due to the complexity comprehension activity requirement, schools should remind students to develop vocabulary knowledge with grammatical and inference ability coordinately on comprehension task performance, enhancing mental image construction via well-constructed deep semantic meaning.

### Limitations and Implications

The current study has four main limitations. First, previous studies reported that vocabulary might have an independent contribution to reading comprehension directly rather through decoding and linguistic comprehension (Ouellette and Beers, 2010; Tunmer and Chapman, 2012); the current study results did not fully support this statement through simple meta-analytic approach. For future studies, a network meta-analytical approach may be a reliable approach to investigate the effect. Second, the current study only examined the interaction effect on the association between vocabulary knowledge and reading comprehension from grade or education stage, language type, and sampling area; the other factors' effect [e.g., text comprehension level (Sparks et al., 2008)] was not included. Third, it did not investigate the interaction effect within selected moderators. Finally, from secondary school stage to Master's stage, all selected studies reported Chinese students' correlation between L2 vocabulary knowledge and L2 reading comprehension only.

The results of the current study indicated the correlation between reading comprehension and vocabulary for Chinese participants, and age or education stage should be considered as a key variable to control due to the significant interaction effect with the target correlation. Second, for those intervention designs that aim to improve reading comprehension through a vocabulary intervention program, the appropriate time for higher intervention effect size should be during primary school, secondary school, and undergraduate stage. Finally, regarding teaching activities, because the contribution of vocabulary on reading comprehension decreased since secondary school, teaching activities should pay more attention to other linguistic factors' (e.g., inference) design during the school reading program.

## Conclusion

This study found the inverted U-shape correlation picture between vocabulary knowledge and reading comprehension in Chinese participants. Results showed that vocabulary knowledge might have an independent effect on reading comprehension in each education stage, which rejected the possible interaction effect of grade group in primary school, sampling area, and language type in different script cognition. Results showed that the correlation effect size decreased since secondary school education stage, the reason being the higher difficult level of text comprehension, which suggested that other higher-order thinking factors (e.g., inference) may contribute a higher proportion on text comprehension.

## Data Availability Statement

The datasets generated for this study are available on request to the corresponding author Yi Tang.

## Author Contributions

YD drafted the most part of the manuscript and did data analysis. YT revised the manuscripts and did data analysis. BW-YC provided critical comments to the draft. WW and W-YD helped data collection and provided comments to the draft. All authors contributed to the article and approved the submitted version.

## Conflict of Interest

The authors declare that the research was conducted in the absence of any commercial or financial relationships that could be construed as a potential conflict of interest.
